# Faecal-wood biomass co-combustion and ash composition analysis

**DOI:** 10.1016/j.fuel.2017.05.038

**Published:** 2017-09-01

**Authors:** Tosin Onabanjo Somorin, Athanasios J. Kolios, Alison Parker, Ewan McAdam, Leon Williams, Sean Tyrrel

**Affiliations:** a Offshore Renewable Energy Engineering Centre, Cranfield University, Cranfield MK43 0AL, UK; b Cranfield Water Sciences Institute, Cranfield University, Cranfield MK43 0AL, UK; c Competitive Creative Design Centre, Cranfield University, Cranfield MK43 0AL, UK

**Keywords:** Faecal ash, Combustion, Fuel blending, Non-sewered sanitary systems, Nano-membrane toilet, Soil conditioner

## Abstract

Fuel blending is a widely used approach in biomass combustion, particularly for feedstocks with low calorific value and high moisture content. In on-site sanitation technologies, fuel blending is proposed as a pre-treatment requirement to reduce moisture levels and improve the physiochemical properties of raw faeces prior to drying. This study investigates the co-combustion performance of wood dust: raw human faeces blends at varying air-to-fuel ratios in a bench-scale combustor test rig. It concludes with ash composition analyses and discusses their potential application and related problems. The study shows that a 50:50 wood dust (WD): raw human faeces (FC) can reduce moisture levels in raw human faeces by ∼40% prior to drying. The minimum acceptable blend for treating moist faeces without prior drying at a combustion air flow rate of 14–18 L/min is 30:70 WD: FC. For self-sustained ignition and flame propagation, the minimum combustion temperature required for conversion of the fuel to ash is ∼400 °C. The most abundant elements in faecal ash are potassium and calcium, while elements such as nickel, aluminium and iron are in trace quantities. This suggests the potential use of faecal ash as a soil conditioner, but increases the tendency for fly ash formation and sintering problems.

## Highlights

• Co-combustion analysis was investigated using a bench-scale combustor test rig.
• Raw human faeces (FC) contained 73.9 ± 4.4 wt% moisture as received basis.
• Blending with wood dust (WD) in a 50:50 ratio reduced moisture levels by ∼40%.
• Minimum acceptable blend for combustion without prior drying is 30:70 WD:FC.
• Fuel burn rates are 3.18–4.49 g/min for all the blends at air flow of 12–18 L/min.
• Oxygen, potassium and calcium are the most abundant elements in faecal ash.

## 1 Introduction

The development of next-generation on-site sanitation facilities is underway in many parts of the world including the United Kingdom [1], [2], Switzerland [3], and Canada [4]. Under the Reinvent the Toilet Challenge (RTTC), a Water, Sanitation & Hygiene program launched by the Bill and Melinda Gates Foundation, toilets are being designed to safely treat human excreta and recover useful by-products such as clean water, fertiliser and electricity. Toilets are required to operate in areas where there may be no water, energy or sewer connections. They must also be affordable: expected to cost no more than $0.05 per user per day [5]. These facilities are intended for the 40% of world’s population that have no access to sanitation and including the 1 in 7 people that rely on unhygienic toilets, unsafely emptied pit latrines or open defecation, particularly where sanitation infrastructures are overburdened or not in existence.

The on-site sanitation facility ‘Nano Membrane Toilet’ (NMT) that is being developed at Cranfield University as part of the RTTC employs processes such as sedimentation and membrane filtration for removing and purifying the loosely bound water (mainly urine) in human excreta, and technologies such as the optimised archimedes screw for transport of residual solids into an energy conversion unit, with subsequent thermochemical conversion of the solids into ash and heat energy [1]. Since raw human faeces contain as much as 77 ± 4 wt% moisture [6] and to limit the moisture entering the energy conversion unit, the residual solids are projected to undergo a pre-treatment process that involves partial drying and pelletisation. These processes can consume a significant part of the energy contained and recovered from the faecal material, if not adequately managed. More so, raw human faeces possess peculiar viscous, “sticky” characteristics, varying water-bound levels, and compositional non-uniformity from un-chewed and undigested foods that make sample homogeneity, handling and use difficult [6]. For instance, the “lumpy” food particles in the residual solids cause blockages and the sticky characteristics promote adherence to mechanical equipment [6], [7], thereby making extrusion and pumping challenging. There is, therefore, a need to upgrade the physiochemical properties of the faecal matter, prior to drying and pelletisation, a process that can be improved by fuel blending.

Fuel blending is a process that combines two or more materials to produce a finished product with superior quality. It is a widely used approach in biomass combustion, particularly for feedstocks with high moisture, ash content (AC) or low calorific value [8]. Direct combustion of biomass and coal is progressively deployed in coal fired plants, particularly in Germany, and the Netherlands [9], [10]. Agricultural waste, municipal solid waste and energy crops are also increasingly applied in boilers and power plants [11] at 10–25% blending ratio with fossil fuel or similar biomass material to reduce emissions and fossil fuel consumption or to utilise waste resources [12], [13]. In developing countries, wood dust — also known as sawdust — is often used as bedding material and for clearing poultry waste in large quantities, and subsequently disposed of via open burning. Therefore, a fuel pre-treatment process that involves a blend with materials such as wood dust can make raw faeces more suitable for thermo-chemical conversion and for use in on-site sanitation technologies. This can enhance sustained fuel ignition and flame propagation, thereby improving process efficiency and the continuous use operation of the energy conversion unit. It can minimise the life-cycle energy requirement for drying and simplify the fuel pre-treatment processes. Overall, this can accelerate product development and diffusion with a link to existing user behaviour.

Co-combustion of two or more biomasses is a well-documented approach in the literature. Barber [10] showed that the addition of 4% dewatered sewage sludge cake and dried pellets was suitable for electricity generation in a 760 MW coal fired power station at a furnace temperature of 1200 °C and fuel feed rate of 240 t/h. There was no adverse effect on emissions and boiler operation, provided the overall moisture content (MC) of the feedstock did not exceed 14 wt% by weight. Stelmach and Wasielewski [14] investigated the co-combustion of sewage sludge with coal in a pulverised coal boiler and uphold the approach for sludge utilisation in Poland. Similar studies are presented for sewage sludge utilisation in circulating fluidized beds [15], [16], [17]. For thermogravimetric analyses, Jin et al. [18] showed that different blending ratios of dewatered sludge and coal had minimal influence on ignition temperature, flammability index, combustion characteristics index and emissions, provided the addition of dewatered sludge did not exceed 20 wt%. It was observed that NOx and SO_2_ emissions reduced with increasing blending ratio, particularly at 30% dewatered sludge. Wang et al. [19] investigated the transformation of nitrogen in different blends of municipal sewage sludge (MSW) and cotton stalk, wheat and corn straw at a heating rate of 100 °C/min up to 900 °C and O_2_ flow rate of 100 mL/min. They observed volatile nitrogen as the predominant form and reduced NOx emission, particularly for 30:70 MSW:corn straw blend. Peng et al. [20] showed that the process improved the char catalytic and alkali-metals melt-induced effect on the decomposition of textile dyeing sludge when the sludge was mixed and heated up to 900 °C at an air flow rate of 50 mL/min with 60 wt% microalgae sludge. Magdziarz and Wilk [21] showed that the co-combustion process favoured the treatment of sewage sludge at varying heating rate conditions, but ignition temperatures were affected by the different blend rates. These studies collectively identify the importance of blending ratios and influence on combustion and/or emission characteristics, but these are specific to the fuel stock.

Beyond energy recovery and emission reduction, fuel blending is being explored for nutrient recovery and utilisation and to understand nutrient transformation in residual ash samples. Li et al. [22] explored the co-combustion of MSW and wheat straw in a vertical tubular furnace reactor, in order to understand the transformation of potassium in ash samples. Yan et al. [23] utilised the method to inhibit the formation of polychlorinated dibenzo-p-dioxins and dibenzofluorans (PCDD/Fsfor) and observed the potential application for hospital waste. Folgueras et al. [24] studied sulphur retention in ash samples recovered from co-combustion of bituminous coal and sewage sludge. The sewage sludge was obtained from a waste treatment plant that utilised FeCl_3_ for coagulation and it was observed that increased sewage addition improved sulphur retention due to the increased calcium oxide content in the sludge. Thus, the blending of the raw faeces with similar biomass material such as wood dust can find good application in on-site sanitation facilities, particularly in rural communities, where wood biomass is a readily available and utilised resource [25].

For faeces-specific studies, there is sparse information on the blending of human faeces with combustible materials for energy generation. Apart from Monhol and Martins [26] who investigated the co-current combustion of dried human faeces with polyethylene waste, other co-combustion studies including those of the authors [6], [28], [29] have primarily focused on the use of 100% human faecal matter or surrogate faecal material [30], [31]. While the co-combustion of faeces with polyethylene waste [26] appears to be viable, the household application of such a fuel blending process can have severe environmental and health implications, due to the potential emissions of persistent organic pollutants such as polybrominated dibenzo-p-dioxins and dibenzofurans (PBDD/Fs), polybrominated diphenyl ethers (PBDEs) and PCDD/Fs [27]. As such, a renewable energy source would be a more ideal blend material. Onabanjo et al. [6] showed that dry human faeces have a comparable energy content to wood biomass with similar combustion temperature profiles. Their study also showed that combustion can be applied to treat faecal matter with up to 60 wt% moisture, provided the combustor temperature is above 600 °C, but raised concern on the direct use of the material due to fuel-peculiar characteristics.

This paper therefore presents the co-combustion of raw faeces with wood dust in a bench-scale combustor test rig, as a means of demonstrating the application of fuel blending in the ongoing development of a novel on-site sanitation technology. Co-combustion experiments were conducted at various blend ratios and air flow rates to determine the minimum blend requirement and the optimum combustion operating conditions. Performance assessment was carried out on the basis of combustion temperature, fuel burn rate, modified combustion efficiency (MCE) and carbon conversion efficiency (η_CCE_). The study concludes with an assessment of the mineral composition of the ash residues recovered from the co-combustion experiments and discusses their potential application and related problems.

## 2 Methodology

### 2.1 Biomass feedstocks

Twenty-eight fresh human faeces were collected anonymously from volunteers at Cranfield University under the approval of the institution’s Research Ethics Committee. These samples were stored at −85 °C for four weeks until collection was complete and thereafter, thawed and mixed for uniform consistency. Blending ratios (10:90, 20:80, 30:70, 40:60 and 50:50) were prepared in triplicates using the mixed stock of raw faeces and wood dust. These are denoted as FC90, FC80, FC70, FC60 and FC50 for wood dust: human raw faeces respectively. The rest of the stock was dried to constant weight in a GENLAB Hot Air Oven at 45 ± 5 °C and denoted as FC100 (100% raw human faeces). The dry 100% wood dust (pelletized) is referred to as WD100.

The ultimate composition, i.e. the relative percentages of carbon (C), hydrogen (H), oxygen (O) and nitrogen (N), was determined using a thermal CHN analyser (Elementar Vario ELIII). The proximate composition, mainly MC, volatile matter (VM) and AC was determined using the protocols described in BS EN 14774-3, BS EN 14775 and BS EN 15148 for MC, VM and AC. The fixed carbon content was calculated by deducting the wt% of MC, VM and AC from 100%. The higher heating value (HHV), a measure of the gross energy content of the biomass feedstocks, was determined using an automatic Parr Bomb Calorimeter. The samples were combusted in oxygen at 30 atm. The instrument was calibrated with 1 ± 0.1 g of benzoic acid.

### 2.2 Experimental set-up

**Fig. 1:**
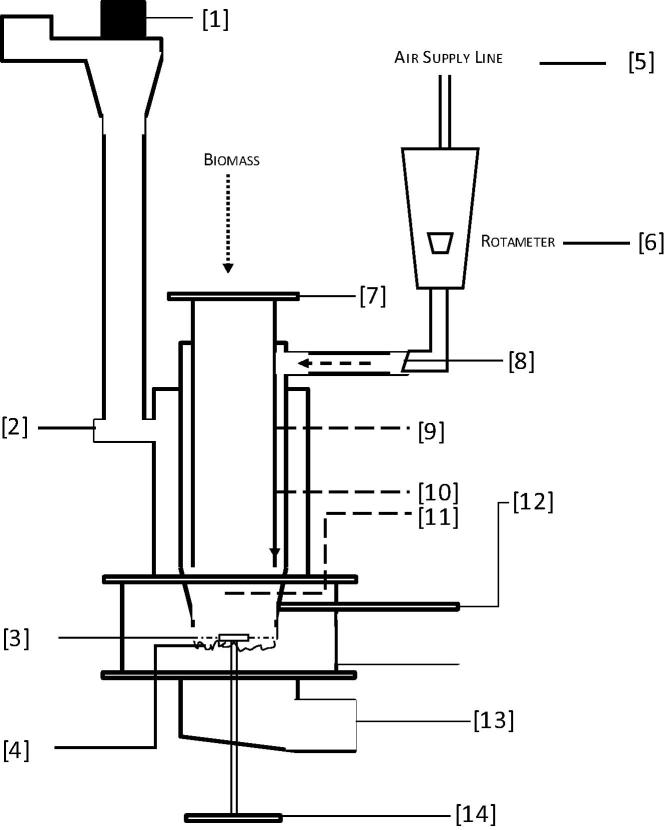
Graphical representation of the bench-scale fixed bed downdraft combustor test rig [6]. (1) Suction Fan, (2) Exhaust Port, (3) Ash Agitator, (4) Fuel Bed (Grated Surface), (5) Air Supply Line, (6) Rotameter, (7) Fuel Inlet Gate, (8) Primary Air Inlet, (9) Upper Combustor Temperature, (10) Lower Combustor Temperature, (11) Combustion (Bed) Temperature, (12) Heater Temperature/Air Igniter, (13) Ash Collector and (14) Ash Rotor.

The detailed description of the experimental test rig is described in previous author’s publication [6]. The initial test rig was procured from RTI International/Colorado State University and modified as part of the Nano-membrane Toilet Phase II project, RTTC. The current view of the test rig is graphically provided in Fig. 1 with summary below.

The downdraft reactor has a volumetric capacity of about 1 L with a solid grate at the bottom that allows ash to pass through to the ash collection system after combustion. The fuel is fed through point 7 and heated initially by passing hot air over the surface of the AC igniter at point 12. After ignition, which is evident by the sudden increase in combustor temperature and change in flue gas composition, the source of the pre-heated air is shut and primary air inlet is open at point 8 for ambient air supply via a rotameter (point 6). The flue gas exits at point 2 into the extraction system. For gas measurements, flue gas is bled at point 2 and analysed for dry gas composition, specifically carbon dioxide (CO_2_), carbon monoxide (CO), methane (CH_4_) and hydrogen (H_2_). This is following the condensation of water vapour and tar removal. Temperature measurements are achieved with K-type thermocouples and the data are recorded every second in a Picolog software environment.

### 2.3 Experimental plan

**Fig. 2:**
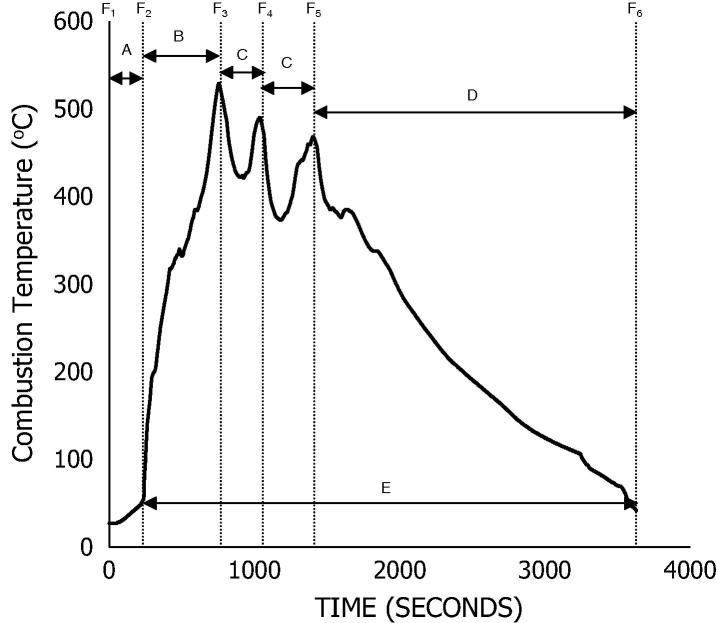
Co-combustion temperature curve as defined in this study. F_1_ -start of experiment and introduction of wood pellet, F_2_ -Ignition point, F_3–5_ -introduction of blended samples or raw dry feedstocks, F_6_ -end of experiment. Zone A -ignition delay period; Zone B -flame propagation period; Zone C -onset of char burn out and final stages of combustion, also devolatilisation and combustion of new fuel; Zone D -complete burn out stage.

At the start of the experiment, 75 g batch of dry wood pellets were burnt in the reactor at a defined air flow rate, corresponding to peak temperatures of 600 ± 20 °C. This initial fuel feed simulates the ignition requirement for the energy conversion unit that is being developed for the NMT and provides a hot bed for burning moist faeces. Following this process, 25 g of wood dust: faeces blends were introduced intermittently — refer to Fig. 2. Different fuel blends ranging from 0 to 50% were tested at air flow rates of 12–18 L/min. These air flow values were selected based on excess air conditions for the various fuels and considering a slightly lean-to-rich air flow regime, which is determined from the theoretical air and stoichiometric air-to-fuel ratio At the end of the test, ash was collected, weighed and analysed for any residual carbon [32]. The elemental composition of the ash samples was determined using a FEI XL-30 Environmental Scanning Electron Microscope (ESEM) that is integrated with an energy dispersive X-ray spectroscopy (EDX) analyser. Measurements were processed with AztecEnergy V2.2 software.

### 2.4 Repeatability and performance calculation

To measure the repeatability of the tests, duplicate combustion tests were conducted for FC50 and WD100 samples. The Root Mean Square (RMS) and coefficient of variance (COV) were determined using Eqs. (1), (2).

(1) $$\text{RMS,}\;\%=\sqrt{\frac{\underset{i}{\sum }{\left({\text{REF}}_{i}-{\text{MOD}}_{i}\right)}^{2}}{\text{D}}}$$

(2) COV=σ/μ

where REF is the value obtained from the first experiment, MOD is the value obtained for the duplicate test, D is the number of data for each of the parameters, i, and σ and μ are the standard deviation and mean for the duplicate tests.

Performance assessment was carried out on the basis of combustion temperature, fuel burn rate, MCE and η_CCE_. MCE (%) is the ratio of carbon dioxide to total carbon dioxide and carbon monoxide in the flue gas, as expressed in Eq. (3) while η_CCE_ is estimated using the percentage of carbon in the flue gas to the carbon in the fuel feed less those in the ash samples —Eq. (4). The fuel burn rate is defined using Eq. (5).

(3) $$\text{MCE,}\%=[{\text{CO}}_{2}]/([{\text{CO}}_{2}]+[\text{CO}])$$

(4) $$\eta_{\text{CCE}}\text{,}\%=((([\text{CO}]+[{\text{CO}}_{2}]+[{\text{CH}}_{4}])\cdot [{\text{M}}_{\text{c}}])\cdot \text{Y})/(\text{C}{\%}_{\text{fuel}}-\text{C}{\%}_{\text{ash}})\cdot {\text{V}}_{i}$$

(5) $$\text{Fuel Burn Rate,}\;\text{g}/\text{min}={\text{M}}_{\text{fuel}}/{\text{t}}_{\left(\text{s}\right)}$$

where C%_fuel_ is the mass percentage of carbon in the fuel as determined by the ultimate analysis, and C%_ash_ is the mass percentage of residual carbon in the ash, [CO], [CO_2_], [CH_4_] are the molar percentages of CO, CO_2_ and CH_4_ in the flue gas respectively. M_C_ as the molecular weight of carbon is 12 kg/kmol, Y is the estimated flue gas yield (Nm^3^/kg), M_fuel_ is the mass of the fuel burned in grams over a duration (t) as in Equation 3. The duration in minutes is taken as the time ignition starts and end of char burn out. V*_i_* is 22.4 L/kmol, the molar volume occupied by ideal gas at standard temperature and pressure (STP).

## 3 Results

### 3.1 Biomass characteristics

The elemental and proximate compositions of the raw feedstocks (WD100 and FC100) are presented in Table 1. The MC values are expressed in weight percentages (wt%) on an as received basis (arb) while the values for VM, FC and AC are expressed as wt% on a dry-weight basis (db). The HHV (MJ/kg) of the raw feedstocks and the MCs, wt% arb of the blended feedstocks are also presented.

**Table 1:** Proximate and ultimate composition of the raw feedstocks (WD100 and FC100).

Samples	Moisture Content (wt% as received basis)	Proximate Analysis (wt% dry basis)			Ultimate Analysis Total (wt% dry basis)				HHV (MJ/kg) (dry basis)
		Volatile Matter	Fixed Carbon	Ash Content	Carbon	Hydrogen	Nitrogen	Oxygen*	
FC100	73.90 ± 4.38	82.00 ± 1.11	0.00 ± 0.00	18.33 ± 1.27	47.12 ± 1.18	6.51 ± 0.40	5.74 ± 0.00	22.30 ± 0.66	23.39 ± 0.13
WD100	9.00 ± 0.18	99.10 ± 0.00	0.08 ± 0.00	0.72 ± 0.00	50.68 ± 1.78	6.64 ± 0.14	0.09 ± 0.03	41.88 ± 0.65	18.12 ± 2.42
FC100	77.60								
FC90	70.40								
FC80	66.50								
FC70	64.70								
FC60	55.00								
FC50	47.60								

FC100- 100% Human Faeces, WD100- 100% Wood Dust; Oxygen*: 100 – (wt% of C, H, N and ash), FC90 -10:90 wood dust: raw human faeces, FC80 -20:80 wood dust: raw human faeces, FC70 -30:70 wood dust: raw human faeces, FC60 -40:60 wood dust: raw human faeces, FC50-50:50 wood dust: raw human faeces.

Table 1 shows that the average MC in the FC100 is 73.9 ± 4.4 wt% arb but that of the wood dust is less than 10 wt% arb. On a dry weight basis, the FC100 and WD100 samples have a VM content of 82.0 ± 1.1 wt% and 99.1 ± 0.0 wt% respectively. While the AC in WD100 sample is <1 wt% db, the AC in FC100 contains nearly one-fifth. As such, the weight percentages of fixed carbon in both samples are negligible. In terms of elemental balance, the FC100 contains 47.12 ± 1.18 wt% (C), 6.51 ± 0.40 wt% (H), 5.74 ± 0.00 wt% (N) and 22.30 ± 0.66 wt% (O). The wood dust on the other hand contains higher amounts of C, H and O with values of 50.68 ± 1.78 wt%, 6.64 ± 0.14 wt% and 41.88 ± 0.65 wt% respectively, but low amounts of N at 0.09 ± 0.03 wt%. Comparing the energy content of the dry faecal matter and wood dust, it is observed that the HHV of the WD100 is 18.12 ± 2.42 MJ/kg while that of the FC100 is 23.39 ± 0.13 MJ/kg. The moisture in the FC100, however, places the HHV of the raw faeces at about 6 MJ/kg.

Comparing the results obtained in this study to those in the literature, the moisture levels for the raw faeces are within the range (63–86 wt% arb) reported in [33]. Previous analysis by the authors [6] also reported an average moisture value of 77 ± 4 wt% while Yadav et al. [34] stated a value of 80 ± 5 wt% for raw faeces. For moisture levels in the wood dust, Nemli et al. [35] and Bana and Banthia [36] reported a moisture range of 3–17 wt% arb, which is similar to the value reported in this study. Depending on wood source and type, the percentages of organic matter (fixed carbon and VM) are reported in excess of 99 wt%, with VM having at least 80 wt% on a dry-weight basis, while AC is reported as less than 2 wt% [28].

For elemental balance, Demirbas [37] reported a percentage range of 49.5–51.9 wt% (C), 6.0–6.2 wt% (H), 40.9–42.4 wt% (O) and 0.3–0.4 wt% (N) for different types of wood biomass. Jenkins et al. [38] also showed a range of 46.3–49.9 wt% (C), 5.39–5.90 wt% (H), 34.5–41.80 wt% (O) and 0.57–0.61 wt% (N). For dry faecal samples, Onabanjo et al. [6] reported C, H, O and N percentages of 51 ± 2 wt%, 7 ± 0 wt%, 21 ± 3 wt% and 4 ± 1 wt% respectively. Their study also showed that the HHV of faecal biomass was 24.73 MJ/kg on a dry-weight basis, a value that is considerable higher than the range of 19.2–20.2 MJ/kg reported for different wood types [39]. Typically, the HHVs of biomass are well documented to be in the range of 14–21 MJ/kg [37], but only a few studies have been undertaken with human faeces.

Unlike coal-wood, -municipal solid waste or -sewage sludge blends where there is constraint on the amount of low calorific fuel that can be added in coal combustion, Table 1 shows that increasing amounts of wood dust improve the physiochemical properties of the raw faeces. The final moisture levels in the blended materials were reduced by 9% (FC90), 14% (FC80), 17% (FC70), 29% (FC60) and 39% (FC50); this is because there is partial absorption of the moisture in the raw faeces by the fibrous wood dust. It is a good indication of application in on-site sanitation technologies, where additional moisture can be expected from unremoved free water that is added into the system, such as urine, flush water or cleaning agent. The similarities of the compositional analysis for the triplicate samples drawn for the fuel feedstocks as presented in Table 1, as well as the close comparison with those in literature confirm a reliable use of the fuel characterisation results.

### 3.2 Co-combustion analysis

Fig. 2 presents the combustion temperature curve with respect to time for the co-combustion of the FC50 sample at an air flow rate of 12 L/min. The diagram reflects points F_1_ to F_6_, which indicate the start and end of each experiment respectively. At point F_1_, 75 g (unless otherwise stated) of wood biomass pellets are introduced into the reactor. This is preceded by fuel ignition (F_2_), the point at which the igniter is turned off and primary air is supplied. At peak temperature (Point F_3_), 25 g of blended fuel are added to the reactor and this is followed by subsequent additions, F_3_ to F_5_. For the time frames, Zone A is taken as the ignition “delay” period, i.e. the time it takes for the solid particles to gain sufficient heat and the onset of ignition. Zone B is taken as the flame propagation period, which involves devolatilisation and combustion. Zone C is the onset of char burnout and the final stages of combustion, also the point at which new fuel goes through a repetitive devolatilisation and combustion process. Each process is allowed to reach a peak temperature before a new fuel is added. Zone D denotes a period of complete burnout for the final fuel added and the residual ash is recovered at the end of the experiment. To allow for consistency of all the tests, a time frame of 1500 s, excluding ignition delay, was considered for fuel addition. This is to demonstrate a short time frame of continuous burn and self-sustained ignition. A test is considered to be a “pass” provided a peak temperature is observed following the addition of the fuel, while the absence of an increase in temperature or a combustor temperature below 400 °C following the addition of the fuel blend is considered a “failed” test. Combustion performance was assessed using MCE and η_CCE_, along with other parameters, such as fuel burn rate and peak combustor temperature.

#### 3.2.1 Influence of air flow rate

**Fig. 3:**
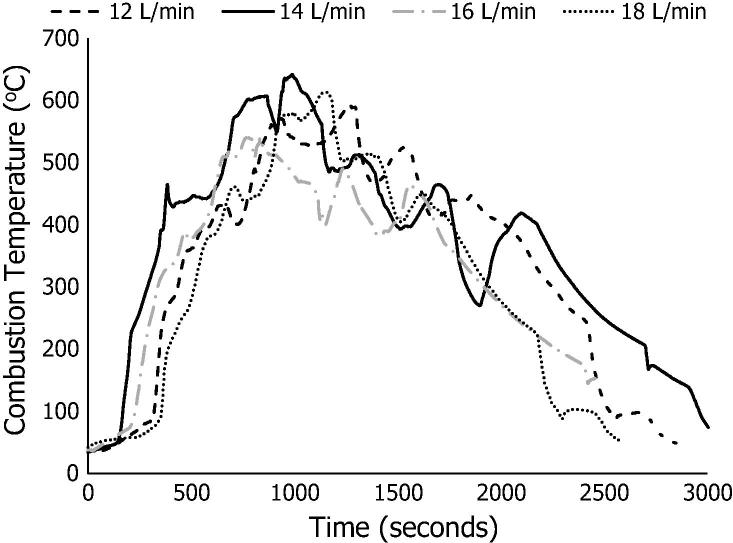
Combustion temperature curves for co-combustion of FC50 at 12–18 L/min.

Fig. 3 presents the combustion temperature curves for the co-combustion of the FC50 sample at air flow rates of 12–18 L/min, while the co-combustion performance analyses for these processes are summarised in Table 2.

**Table 2:** Co-combustion performance for FC50 at 12–18 L/min air flow rates.

Samples	Air Flow Rate (L/min)	Fuel Burn Rate (g/min)	Carbon Conversion Efficiency (%)	LHV_gas_	MCE (%)
FC50	12	3.45	91.0	0.245	89.3
	14	3.71	97.5	0.228	89.8
	16	4.24	69.6	0.193	90.5
	18	5.02	59.5	0.144	90.7

Fig. 3 shows that the highest combustion temperature (642 °C) was reached at an air flow rate of 14 L/min, while the lowest (541 °C) was observed at 16 L/min. The rest of the temperatures averaged at ∼ 600 ± 10 °C. These temperatures were achieved after the first introduction of the blended FC50. The ignition delay was slightly longer at 12 L/min and 18 L/min, with values of 5.27 min and 5.43 min respectively, but minimal for the rest of the air flow rates: 2.53 (14 L/min) and 3.30 (16 L/min).

Table 2 shows that the carbon conversion efficiencies decreased from 91.0% to 59.5%, which corresponds to the increase in air flow rate from 12 L/min to 18 L/min. Fuel burn rates had a linear relationship with air flow rates. The lowest fuel burn rate was 3.45 g/min at 12 L/min and the highest was 5.02 g/min at an air flow rate of 18 L/min. The MCE, which is the ratio of carbon dioxide to total carbon dioxide and carbon monoxide in the flue gas, varied narrowly between 89–90% and the estimated total LHV of the gas was at most 0.25 MJ/kg for the different air flow rates.

#### 3.2.2 Influence of blending ratio

**Fig. 4:**
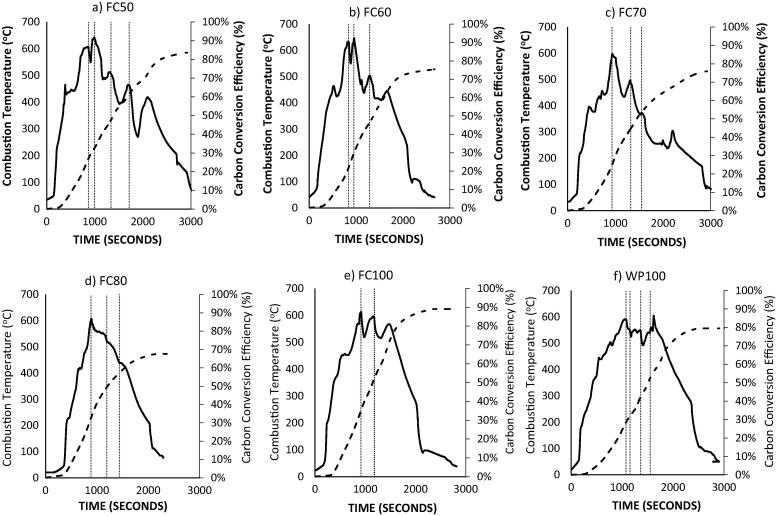
a–f: Combustion temperature curves and the cumulative carbon conversion efficiencies for combustion of (a) FC50, (b) FC60, (c) FC70, (d) FC80, (e) FC100 and (f) WD100 at air flow rate of 14 L/min.

Fig. 4a–f presents the combustion temperature curves and the cumulative carbon conversion efficiencies for the combustion of the raw feedstocks (WD100 and FC100) and their blends at an air flow rate of 14 L/min. The results show that the co-combustion of raw human faeces and wood dust is attainable at a combustion temperature of 600 ± 20 °C for all the different blends. While the processes for the FC50, FC60 and FC70 contributed sufficient heat to ignite a new batch of moist fuel (25 g), there was a continuous drop in combustion temperature for the FC80 fuel blend. At the initial combustor temperature of ∼500 °C, the combustion of FC50 and FC60 samples produced sufficient heat to treat a new batch of fuel; however, the FC70 sample had just enough to convert the fuel to ash. At temperatures between 400 °C and 450 °C, the FC50 blend was the only ideal candidate.

The co-combustion performance analysis of the blended fuels in Table 3 shows an increasing conversion of the fuel with an increasing blend with wood dust. Here, carbon conversion efficiencies of 83.7%, 76.1%, 77.1% and 69.2% are observed for FC50, FC60, FC70 and FC80 samples respectively at an air flow rate of 14 L/min. The fuel burn rates also follow a similar order with values ranging from 3.75 g/min (FC50) to 3.18 g/min (FC70) with the exception of the FC80 sample where an increase is observed. The MCEs of the FC50 and FC60 samples are ∼ 90% but much higher for the moister fuels — 95.5% (FC70) and 87% (FC80). For the combustion of the raw feedstocks however, the fuel burn rates are 3.77 g/min (FC100) and 4.2 g/min (WD100), the carbon conversion efficiencies are 86.1% (FC100) and 79.5% (WD100), while the MCEs are 82.7% (WD100) and 79.3% (FC100).

**Table 3:** Co-combustion performance analysis for the blended fuels.

Samples	Air Flow Rate (L/min)	Fuel Burn Rate (g/min)	Carbon Conversion Efficiency (%)	LHV_gas_ (MJ/kg)	MCE (%)
WP100	14	4.20	82.7	0.46	82.5
FC50		3.75	83.7	0.23	89.8
FC60		3.73	76.1	0.17	89.9
FC70		3.18	77.1	0.08	95.5
FC80		4.07	69.2	0.21	87.0
FC100		3.77	86.1	0.45	79.3
WP100		4.48	79.7	0.33	84.1
FC50	16	4.49	75.8	0.26	86.7
FC60		3.91	75.8	0.17	89.8
FC70		3.59	71.6	0.11	93.4
FC80		2.75	65.1	0.11	91.5
FC100		3.55	84.6	0.28	83.9
WP100		4.51	85.9	0.44	84.0
FC50	18	4.42	88.5	0.55	82.2
FC60		4.12	69.4	0.14	90.7
FC70		4.11	63.1	0.12	91.5
FC80		3.37	64.0	0.11	91.5
FC100		4.04	78.4	0.29	86.0

Similar trends are observed at the air flow rates of 16 L/min and 18 L/min for fuel burn rates and carbon conversion efficiencies. The fuel burn rate and carbon conversion efficiency reduced slightly with moister fuels. The main differences with the 14 L/min scenario include: a) higher fuel burn rate for all the blended fuels at 16 L/min and 18 L/min, although the values were much higher at 16 L/min, b) increasing fuel burn rates for the WD100 sample with a reducing fuel burn rate for the FC100, except at 18 L/min, and c) lower carbon conversion efficiencies for the FC100 and WD100 samples at higher air fuel rates. The MCE varied from 86.7–93.5% for the 16 L/min and 82.2–91.5% for 18 L/min. For the FC80 samples, all the results were mostly an exception as there was no flame combustion as a result of addition of the blended sample. The heat recovered from the initial fuel feed was used for converting the samples under smouldering ‘low temperature’ conditions. The results for the FC90 samples for all the air flow rates are regarded as failed tests and not presented because there was no fuel ignition in all the test cases.

**Fig. 5:**
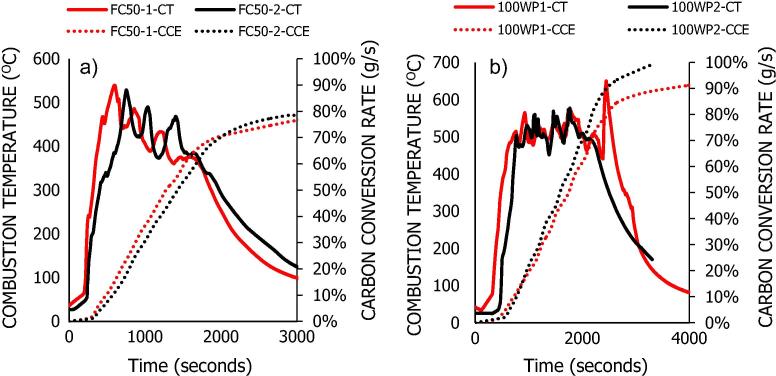
a-b: Repeated combustion analysis for (a) FC50 and (b) WD100. Temperature curves (CT) and the cumulative carbon conversion (CCE) efficiencies as a function of time and at air flow rate of 12 L/min.

The COVs and RMS errors for the different combustion performance parameters as obtained from the duplicate combustion tests of the FC50 and WD100 samples at combustion air flow rate of 12 L/min with initial feed of 50 g dry wood pellets are presented in Table 4. The combustion temperature curves and the corresponding carbon conversion efficiencies for both duplicate tests are presented in Fig. 5a-b. The COV was at most 0.02 for the different combustion performance parameters for the FC50 samples, and varied between 0.03–0.08 for the WD100 samples. Using RMS, the percentage error is <0.15% for the different parameters. These values show that the tests are quite repeatable. Furthermore, Fig. 5a-b confirms the similarities in the combustion temperature curves and the corresponding carbon conversion efficiencies. The spike combustion temperature observed for the WD100 at the tail end of the analysis could be as a result of hot fuel material passing across the fuel grate and close to the thermocouples. Typically, combustion performance would vary with reactor design, operating conditions, fuel physical properties and feeding systems; however, the results presented in this study provide a reliable close estimate.

**Table 4:** Repeated co-combustion performance analysis for FC50 & WD100 at 12 L/min.

Samples	Air Flow Rate (L/min)	Peak Combustion Temperature (°C)	Fuel Burn Rate (g/min)	Carbon Conversion Efficiency (%)	MCE (%)
FC50-1	12	538	2.98	81.2	88.4
FC50-2		524	3.03	78.4	91.3
WD100-1		650	3.93	91.2	86.6
WD100-2		577	4.21	99.2	82.9
COV-FC50		0.02	0.01	0.02	0.02
COV-WD100		0.08	0.05	0.06	0.03
RMS ERROR		37	0.14	0.04	0.02

#### 3.2.3 Residual ash analysis

**Fig. 6:**
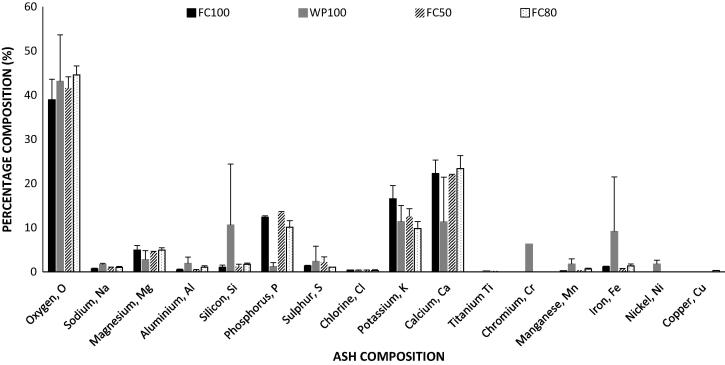
Ash composition analysis for the raw feedstocks and some of the blended fuel.

The elemental composition of the ash recovered from the experimental test rig after combustion of the raw feedstocks and some of the blended samples are shown in Fig. 6. The results show that K and Ca are the main elements in all of the ash samples, apart from the major contributions of oxygen. While the compositions of K and Ca are as high as 16.53 ± 3.01% and 22.26 ± 3.03% in the 100% faecal sample respectively, these values are much lower in the 100% wood dust — 11.33 ± 3.66% (K) and 11.32 ± 10.10% (Ca). Elements such as nickel, copper, titanium, manganese, iron, aluminium, silicon, sodium and chromium are present either in trace amounts or absent in the FC100 and their blends, while the WD100 contains as much as 9.14 ± 12.33% (Fe), 6.30 ± 0.00% (Cr) and 10.59 ± 13.83% (Si) with small amounts of aluminium, titanium, nickel and manganese. The compositions of Mg and P are 4.93 ± 1.02% and 12.38 ± 0.29% respectively, as opposed to 2.77 ± 2.03% (Mg) and 1.20 ± 0.87% (P) in the wood dust. The chlorine composition is relatively similar in both feedstock samples and sulphur compositions vary from 1.36 ± 0.10% and 2.39 ± 3.41% for FC100 and WD100 respectively. The above compositions exclude carbon, but based on loss on ignition method, the unburnt carbon in the FC50, FC60 and FC70 ash samples are <10% and up to 53% in the FC80 sample. For the blends (FC50 and FC20), all the compositions are either similar to the raw feedstocks or reflect the mixture of the two materials.

The review presented by Rose et al. [33] showed that several metals are present in human faeces. The range reported for P, K, Na, Ca and Mg are 1.77–9.86 g/kg, 1.78–4.94 g/kg, 0.80–4.94 g/kg, 2.68–4.27 g/kg and 0.93–2.86 g/kg while those reported for Cu, Fe, Pb, Zb, Ni, Cr and Cd are 6.8 mg/kg, 200 mg/kg, 0.12–6.38 mg/kg, 48.46–67.49 mg/kg, 1.15–1.52 mg/kg, 0.31–0.91 mg/kg and 0.27–6.39 mg/kg respectively. Yadav et al. [34] also reported similar elements; however, higher values are observed for some of these: 11.0 ± 2.0 mg/g (P_2_O_5_), 28.0 ± 1.7 mg/g (K_2_O_5_), 32.0 ± 6.0 mg/g (Ca), 8.2 ± 1.5 mg/g (Mg) and 8.5 ± 1.3 mg/g (Na). Trace elements include Mn (0.27 ± 0.5 mg/g), Zn (0.24 ± 0.04 mg/g) and Ni (0.009 ± 0.002 mg/g). These values confirm that K, Ca, Mg and P are the most abundant elements in human faeces while elements such as Mn, Ni, Cu, Al, and Fe are in trace quantities when they are present.

## 4 Discussion

### 4.1 Application of fuel blending in on-site sanitation technologies

Human faeces are a by-product of digestion, thus contain a blend of undigested food particles, microbial biomass, digestive juices, gut secretions, unabsorbed soluble and insoluble nutrients, fats, protein, water and polysaccharides, as well as cell shedding and mucus from the intestinal lining of the gastrointestinal tract [33]. Wood dusts on the other hand are a by-product of mechanical wood processing. They are generated in large quantities during forest and sawmill operations and in the manufacture of plywood and particleboard, along with other wood wastes including wood cuttings, planer shavings and sander dust [40]. Although wood dusts and human faeces originate from different sources, they have a similar proximate composition and energy value, but are dissimilar in MC and AC, as shown in this study. These contrasting compositions enhance the synergy between the materials. This is advantageous for onsite sanitation technologies because it improves the energy conversion processes of the faecal material and reduces the energy requirements for drying. It promotes the use of materials for energy generation that could otherwise be considered as waste and disposed into the environment or burnt openly in air.

The results in Section 3.2.2 give an indication of the minimum acceptable blend required for combusting the moist faeces without prior drying. They also highlight the minimum combustor temperature range required for a self-sustainable process. At air flow rates of 14–18 L/min, the minimum acceptable blend was FC70, which involves a combination of 30:70 wood dust: raw human faeces. The blend requires a minimum combustion temperature of ∼400 °C for conversion of the fuel to ash and for heat release into the combustion environment. A 50:50 blend of wood dust and raw human faeces reduced the moisture levels in the raw human faeces by ∼40%. To this end, fuel blending provides energy savings but the magnitude of benefits will depend on the moisture levels in the feedstocks. These conditions are not necessarily the optimum conditions for the self-sustained operation of the energy conversion unit. They do, however, define the minimal requirements to overcome the moisture in the raw sample without drying and pelletisation, and the minimum temperature that the combustor needs to be operated at, if the sample were to be fed into the reactor directly as a blend. Below these minimum conditions, the reactor will not be operational, as there is an insufficient hot bed to dry the incoming fuel.

The minimum blending ratios and corresponding minimum combustor temperature are applicable for various scale of operations, but subject to feedstock availability. In many developing economies, wood dusts are readily available and in substantial quantities as waste from wood processing industries. This can ensure a secure feedstock for household-scale toilet operation but availability can be constrained for large-scale applications, if there are competing demands for wood dust for the manufacture of particleboard or for power generation.

Previous thermodynamic analysis by authors [28], [29] have already shown that a high equivalence ratio of >0.55 would be required to drive the endothermic processes of water vaporisation in samples with a high MC of >50 wt%, but equivalence ratio in excess of stoichiometric would be ideal. Based on the elemental composition of the WD100 and FC100, the stoichiometric air supply required for converting the dry wood dust is estimated to be 12.11 L/min and 13.89 L/min for the dry 100% human faeces. The air flow rates of 12–18 L/min therefore correspond to a range of lean-to-rich air supply. For the combustion of the WD100 sample, the lean air supply is 1% at 12 L/min while the excess air supplies are 20% (14 L/min), 32% (16 L/min) and 49% (18 L/min). For the combustion of the FC100 sample, the air flow rates correspond to a lean air supply of 14% (12 L/min) and the excess air supplies are 1% (14 L/min), 15% (16 L/min) and 30% (18 L/min). These estimations suggest that an air flow rate of 14 L/min and 16 L/min is the most suitable operating condition for the conversion of the WD100 and FC100 samples respectively. The co-combustion experiments also confirm these trends and highlights the optimum combustion air-to-fuel ratio (AFR) for converting the partly moist blended samples.

The 14 L/min was the most suitable air flow rate for converting the blended samples because it is slightly above stoichiometric condition and provides a sufficient amount of air for maximum conversion of the fuel — as observed from the carbon conversion efficiency of the FC50 sample in Table 2. It also allows sufficient time for fuel combustion because of the relatively slow fuel burn rate, thereby enhancing combustion efficiency. The heat given off from the process is retained, hence a combustion temperature in excess of 600 °C is observed in Table 2, Table 3.

These results can further be explained using conductive, convective and radiative heat transfer, although faeces drying is a poorly understood process. In the reactor, the partly moist fuel material comes in contact directly with adjacent burning particles and are enclosed by hot air gases. For these particles to attain ignition point quickly, they must absorb heat from the surrounding gasses, a process that is however limited for the partly moist blended fuels and results in relative low combustion temperatures, due to the presence and release of fuel moisture. To enhance fuel ignition and flame propagation, an optimum air supply of 14 L/min is supplied to facilitate drying and combustion, processes that are governed by flame temperature, residence time and turbulence of the gas mixtures [41]. As such, there is increased residence time and relative slow fuel burning rate. The condition then overcomes the evasive energy requirement for drying and achieves complete combustion. At 16–18 L/min however, the heat loss in the excess air exceeds the heat recovered from the clean efficient burn and so carbon conversion efficiency reduces significantly and combustion temperature drops rapidly. At 12 L/min, the air flow is just slightly below stoichiometric but the evasive energy absorbed by water evaporation reduces process efficiency.

These air flow regimes are best suited for small-scale applications, but similar operating range can be established for large-scale systems, provided the optimum air-to-fuel ratio are maintained, all of which depend on treatment requirement. The NMT is a domestic-scale facility designed to operate for a household of ten people with estimated total solids of 50 ± 12.5 g/cap/day, as such there is a need to treat about 500 g of solids every day. For this application, a slow progressive burn rate and high carbon conversion efficiency are the best criteria for selecting the optimum air flow rate for treating the faecal material. The material can be fed into the reactor using batch, intermittent or continuous feed operation. For an intermittent feed of 2–3 h, the unit will require to operate at a low fuel burn rate of 2.78–4.16 g/min to achieve continuous self-sustained operation. For continuous operation, the reactor will require to treat at least 0.34 g/min of dry solids daily while a batch unit operation can burn as much as the operating and material limits of the reactor. Thus, the fuel burn rates of 3.18–4.49 g/min presented in this study for air flow rates of 12–18 L/min are suitable for an intermittent feed operation, but energy will be required for re-igniting the unit. A continuous feed operation will only require the initial energy supply for ignition and the operation could last from several hours to as long as needed. The high MCEs (>80%) and the low LHVs of the flue gas at all flow rate conditions confirm that the processes in this study are within the combustion range. The high carbon conversion efficiency and the efficient burn ensures optimal recovery of the energy in the fuel and clean gas is produced. To achieve further efficient burn, the fuel conversion processes can be improved by optimising the pelletiser and dryer such that the material has a consistent particle size and shape. Such further fuel preconditioning can improve interactions between the fuel and air. Previous studies [6] have shown that a relative large fuel size (20 mm × 20 mm) can cause the combustion air and gasses to exit the combustion zone earlier than required while small fuel size (5 mm × 5 mm) can cause oxygen starvation and low combustion temperature. Factors such as the opening/closing of the fuel lid to introduce new fuel as well as quantity and the rate of feed also have added influence. Stable combustion temperature and consistent heat release rate can therefore be achieved by ensuring that the fuel particles are fed at optimum conditions, perhaps via an automated fuel feed system. This can further be moderated with optimum air supply and ensures that the particles absorb heat and reach their ignition temperatures quickly.

In view of the findings from this study, an energy self-sufficient micro-combustor is being developed at Cranfield University for continuous treatment of faecal material and blends. This includes the design of a control system to ensure the self-sustained operation of the NMT by monitoring the fuel-to-air ratio, combustor temperature and exhaust gas emissions. Further work will also examine the role of environmental factors on the combustion processes. The successful development of the NMT can contribute significantly to the 2030 Sustainable Development Goals of the United Nation Development Programme. This includes improved sanitation for the one-third of world’s population that currently do not have access to modern toilet facilities. Furthermore, energy contributions for the 1.3 billion people worldwide that have no access to modern energy, especially low-income rural communities with limited access to grid power-solutions. Overall, it can minimise environmental pollution and land degradation that results from open burning of wood wastes or those resulting from open defecation and emptying of latrines in water courses.

### 4.2 Potential applications and problems of faecal ash

The valuable elements in biomass, such as potassium and phosphorus, make faecal ash a potential candidate for fertiliser production and as a soil conditioner [42], [43], especially for low nutrient soils. Contaminants and heavy metals, which have environmental and health implications, are relatively low, absent or in trace quantities; as such, there can be limited concerns on the bio-accumulation and leaching potential of toxic elements. Since potassium and phosphorus are essential elements for nutrient synthesis and uptake [43], as well as important for retaining water in plant cells, the application of faecal ash can influence crop yield and quality. The trace elements can also provide essential micro-nutrients to the plant [44]. In acid-prone soils, ash can be used to adjust pH and improve soil physiochemical properties [45]. But, the high calcium content in the faecal ash can pose the risk of increasing soil pH or altering soil microbiome and any soil-plant-microbe interactions [46]. Some studies have claimed that the nutrients in biomass ash have poor solubility, which in turn limits accessibility to plants [47]. There are also concerns that the P in biomass can leach into surface waters and cause eutrophication due to the agricultural use of those waters [48].

In energy conversion units, ash composition also has its implications. Iron, which exists in the form of oxides, sulphides, carbonates or pyrites can interact with alumina-silicates or other silicate minerals including Ca and K [49]. These components can also interact with functional groups, such as oxygen, sulphur, chlorine and nitrogen, to form large, complex agglomerates that contribute to fouling, corrosion, deposit formation, erosion and slagging [50]. Components such as K and Na are known to reduce ash fusion temperatures, which promote ash deposition along the walls of the reactor. Interactions with Ca or Mg increase the ash melting temperatures due to the formation of Ca- or Mg-silicates, but further interaction with phosphorus can reduce those temperatures [49], [50], [51], [52]. Therefore, complex interactions between the inorganic materials in the ash can influence combustion characteristics, though they do not take part directly or contribute to the heat of combustion. All these interactions depend on fuel origin and handling as well as combustor operating conditions. In this study, the relatively high Ca and Mg, low Si, S, Fe and Cl content in the faecal biomass therefore suggests the potential use of faecal ash as a soil conditioner. High potassium, however, poses the risk on sintering problems and the tendency for fly ash formation [53]. For combustion characteristics, the high AC in faeces retains heat and increases the flow of oxidants due to its porous characteristics. This in turn increases the residence time of the fuel and carbon conversion efficiency. A high accumulation of ash can however, hinder fuel combustion due to air flow disruption.

## 5 Conclusion

This study investigated the co-combustion performance of raw faeces and wood dust in a bench-scale combustor test rig at various blending ratios and air flow rates. The results show that the average MC in the raw faeces is more than two-thirds of the sample but that of the wood dust is less than 10 wt% arb. The contrasting compositional characteristics, however, position the blending of both materials at an advantage. A blend of 50:50 wood dust: raw human faeces can reduce moisture levels in raw human faeces by ∼40% prior to combustion. The minimum acceptable blend for treating moist faeces without prior drying at an air flow rate of 14–18 L/min is 30:70 wood dust: raw human faeces. The minimum combustion temperature required for self-sustainable operation and flame propagation, and ultimate conversion of the fuel to ash is ∼400 °C. Below these minimum conditions, the reactor will not be operational, as there is an insufficient hot bed to dry the incoming fuel and ensure heat release into the system. The most abundant elements in faecal ash are potassium and calcium. Furthermore, magnesium and phosphorus are common elements, while elements such as manganese, nickel, copper, aluminium and iron are in trace quantities when they are present. This suggests the potential use of faecal ash as a soil conditioner, but increases the tendency for fly ash formation and sintering problems.
